# Design of a Full-Range Capacitive Sensor Extensometer Using a High-Precision Ultrasonic Motor

**DOI:** 10.3390/s25041012

**Published:** 2025-02-08

**Authors:** Chen Dou, Wenbo Wang, Hong Li, Yunkai Dong, Weiwei Zhan, Liheng Wu, Jiaxin Chen

**Affiliations:** 1 Ministry of Emergency Management, National Institute of Natural Hazards, Beijing 100085, China; douchen22@mails.ucas.ac.cn (C.D.); lhstress@126.com (H.L.); dongyunkai2012@163.com (Y.D.); hank116@126.com (W.Z.); smiling-lily8013@163.com (L.W.); cjx1090402764@163.com (J.C.); 2 School of Emergency Management Science and Engineering, University of Chinese Academy of Sciences, Beijing 100049, China

**Keywords:** extensometer, terrain deformation observation, ultrasonic motor, capacitive sensor, measurement range extension, PID control

## Abstract

Extensometers are critical instruments for accurately measuring small displacements in terrain deformation monitoring. Conventional extensometers often employ eddy current displacement sensors or differential transformer sensors, which are constrained by structural limitations that hinder their ability to meet high-precision requirements. The capacitive micro-displacement sensor has a high precision of up to 0.1 µm, but it is typically limited by its measurement range, making it unsuitable for directly capturing rapidly changing geological phenomena such as earthquakes and landslides. This range limitation can result in exceedance and measurement errors, severely compromising the reliability and timeliness of the data. To address these challenges, this study introduces a novel design for a full-range capacitive sensor extensometer powered by a high-precision ultrasonic motor. The system integrates an ultrasonic motor with high-sensitivity capacitive sensors, enhanced by a grating scale and PID control algorithms. By enabling real-time signal processing and adaptive correction, the proposed design ensures a wide measurement range while significantly improving the measurement stability and accuracy. Laboratory experiments and field validations confirm the extensometer’s performance, achieving a resolution of 2.0 × 10^−11^ strain, a linearity of 0.024%, and a calibration repeatability of 0.06%. These results meet the stringent requirements of terrain deformation observation and establish the extensometer as a robust solution for micro-displacement measurements. This innovative design enhances the reliability of terrain deformation monitoring and contributes to the advancement of rock mechanics observation technologies.

## 1. Introduction

Micro-displacement measurement is critical for terrain deformation observations and plays a pivotal role in geological disaster monitoring, engineering safety assessments, and environmental change studies [1,2]. Advances in sensor technology have led to the development of measurement instruments with enhanced performance. For instance, J. Corominas and colleagues in Spain employed wire extensometers in the eastern Pyrenees and the Dolomites [3], while M. A. Alias and collaborators in Malaysia developed an extensometer system based on fiber Bragg gratings [4]. In China’s geological monitoring network, numerous micro-displacement measurement instruments have been deployed to acquire real-time terrain deformation data [5,6,7]. For example, the China Seismic Network Center uses borehole strain gauges and other underground equipment to monitor the geological strain at stations in Menyuan and Huangyuan, Qinghai [8,9].

**Figure 1:**
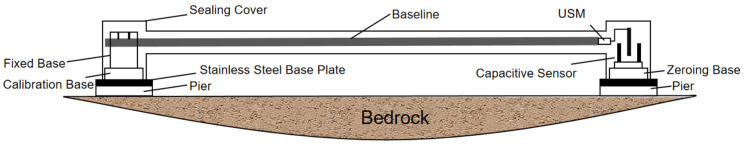
Schematic diagram of the extensometer structure.

Extensometers are precision instruments designed to measure the relative horizontal displacement between two points in crustal rock [10]. These devices frequently employ differential capacitive micro-displacement sensors, offering resolutions as high as 10^−9^ strain [11]. This capability allows extensometers to capture solid tidal and coseismic responses within the crust, making them widely used in geophysics, geodesy, and seismology. A schematic diagram of an extensometer is shown in Figure 1 [12].

Currently, the extensometers deployed in China utilize sensors such as eddy current micro-displacement sensors, differential transformer displacement sensors, and differential capacitive displacement sensors [13]. These sensors offer measurement accuracies ranging from micrometers to nanometers [14], enabling the effective monitoring of solid earth tides and minor crustal deformations. The principles and performance characteristics of these sensor types are summarized in Table 1. Capacitive micro-displacement sensors, due to their superior accuracy, are the preferred choice for terrain deformation monitoring.

Although capacitive sensors offer high precision (up to 0.1 µm), their measurement range is constrained by the electrode gap. High-sensitivity displacement sensors typically cover ranges from 0.1 to 0.6 mm. The maximum horizontal displacement of the semi-diurnal tide to be monitored falls within 1~5 × 10^−8^. To reliably record solid tide movements, extensometers generally require measurement precisions between 5 × 10^−10^ and 10^−9^ strain. Overextended deployments in caves, cumulative drift, and sensor displacement can push the device beyond its linear range, highlighting the limitations of capacitive sensors [15].

To address these constraints, stepper motors are often used to adjust capacitive sensors by driving the middle electrode through mechanisms such as threaded screws [16]. However, stepper motors have limited precision and require auxiliary devices to enhance accuracy. Furthermore, the structural design of existing extensometers introduces challenges in controlling the force exerted by the motor, which can damage or misalign the electrodes. Mechanical instability exacerbates these problems, especially lateral shifts. In such cases, continued motor operation may lead to collisions between electrodes, causing structural damage. The recovery from such incidents often requires external power or manual intervention, increasing system downtime and reducing operational efficiency.

To overcome these challenges, this study presents a full-range capacitive sensor extensometer utilizing a high-precision ultrasonic motor. In this design, an STM32 microcontroller serves as the control unit, paired with a grating scale for position feedback. By driving the motor to precisely control the electrode positioning, the system extends the measurement range while avoiding electrode contact and damage. This approach addresses the limitations of traditional designs, which often require frequent sensor replacements due to the range restrictions. The electric drive mechanism ensures stable operation across an expanded range, reducing maintenance frequency and costs.

Additionally, the inclusion of a grating scale for position feedback allows the real-time monitoring of the electrode positions. The scale’s high resolution facilitates the detection of minute displacement changes, ensuring reliable and precise data acquisition. By replacing the stepper motor-based mechanisms with ultrasonic motors, this design significantly improves the accuracy and mitigates the issues related to zero calibration and equipment wear, marking a notable advancement in extensometer technology.

## 2. Design of Capacitive Sensor Based on Ultrasonic Motor

**Figure 2:**
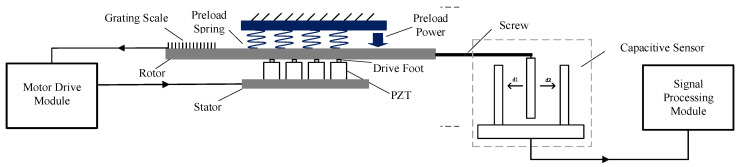
Schematic diagram of the capacitive sensor based on the ultrasonic motor.

The differential capacitive micro-displacement sensor, integrated with a high-precision ultrasonic motor for positioning, consists of four main components: the driving module, the ultrasonic motor, the grating scale, and the capacitive sensor (Figure 2). The driving module applies an alternating voltage to piezoelectric ceramics, inducing frequency mechanical vibrations that drive the rotor movement. A microcontroller generates a frequency PWM signal (2 kHz to 10 kHz) to serve as the input for the piezoelectric ceramics [17]. A power amplification circuit boosts the signal to a voltage sufficient to generate significant mechanical vibrations [18]. A half-bridge driving circuit manages the motor rotation—either forward or reverse—by controlling the conduction timing of two switching transistors.

The microcontroller coordinates all of the components, adjusting the amplitude and frequency of the driving signals to achieve precise control of the ultrasonic motor. The motor itself is a stick–slip linear motor featuring a stator with piezoelectric ceramic vibrating plates and driving feet and a slider composed of metal or ceramic. When the piezoelectric ceramics receive the input signal, they produce frequency vibrations. These vibrations generate axial thrust via stick–slip interactions with the slider, resulting in the linear movement of the motor.

The unique crawling motion of the ultrasonic motor reduces the physical wear caused by friction, significantly extending its operational lifespan. Additionally, this motion characteristic mitigates the excessive thrust that could otherwise damage the electrode plates or screws when the sensor operates near or beyond its limits.

### 2.1. Introduction to Differential Capacitive Sensors

Micro-displacement sensors are precision devices capable of detecting displacement changes at the micrometer or even nanometer scale. These sensors play a vital role in precision applications such as semiconductor manufacturing, MEMS fabrication, and nanomanipulation, where measurements at resolutions down to 0.01 nanometers are required.

**Figure 3:**
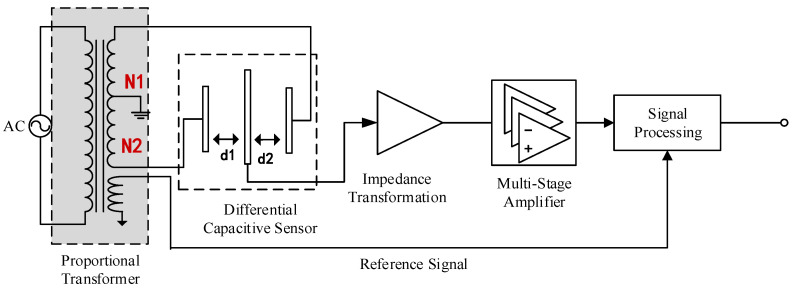
Schematic diagram of the differential capacitive sensor and signal processing circuit.

Differential capacitive displacement sensors are among the most commonly used technologies for high-precision displacement detection. These sensors consist of three parallel metal plates forming two differential capacitors, with a movable electrode positioned between two fixed electrodes [19]. When the baseline is compressed or stretched, the distance between the plates changes, altering the capacitance values. Figure 3 illustrates the structure of the differential capacitive sensor and its signal processing circuit [20].

The signal processing circuit includes modules for AC excitation, a ratio transformer, the differential capacitive sensor, impedance transformation, multi-stage amplification, phase-sensitive detection, and low-pass filtering. The ratio transformer and the differential capacitive sensor together form an AC bridge. Under balanced conditions, the middle electrode plate is in a neutral position and the two half-bridges are equal. Any displacement of the middle electrode plate disrupts this balance, resulting in an output voltage signal [21].

The unbalanced signal from the bridge undergoes impedance transformation, signal amplification, phase-sensitive detection, and low-pass filtering before being transmitted to the AD acquisition unit for the subsequent processing.

When the movable electrode plate approaches the left fixed electrode plate, capacitance *C*_1_ increases, while capacitance *C*_2_ with the right fixed electrode plate decreases [22]. This differential capacitive structure creates a relationship where the capacitance difference Δ*C* = *C*_1_ − *C*_2_ changes in proportion to the displacement of the movable electrode plate. At the initial position, *d*_1_ = *d*_2_ = *d*_0_, the initial capacitances are equal. When the middle electrode plate shifts left by Δ*d*, the distances become *d*_1_ = *d*_0_ − Δ*d* and *d*_2_ = *d*_0_ + Δ*d*. The total relative change in capacitance can be expressed as follows:

$$\frac{\Delta C}{C_{0}}=\frac{\Delta C_{1}-\Delta C_{2}}{C_{0}}=2\frac{\Delta d}{d_{0}}[1+{\left(\frac{\Delta d}{d_{0}}\right)}^{2}+{\left(\frac{\Delta d}{d_{0}}\right)}^{4}+\ldots ]\approx 2\frac{\Delta d}{d_{0}}$$

From the above formula, it is evident that the capacitance difference Δ*C* is directly proportional to the displacement Δ*d* of the movable electrode plate. This proportionality ensures a high degree of linearity, enabling the precise measurement and straightforward interpretation of the displacement based on the changes in capacitance.

### 2.2. Motor Drive Circuit Design

The motor drive circuit utilizes the STM32F103ZET6 microcontroller from STMicroelectronics as the primary control unit. This microcontroller is characterized by its high processing speed, low power consumption, robust performance, and excellent scalability, making it well-suited for diverse measurement and control applications. The control system generates an 80 MHz clock using an external crystal oscillator and produces a PWM signal with a duty cycle ranging from 0% to 20% at a frequency of 625 kHz, which serves as the motor’s input signal.

**Figure 4:**
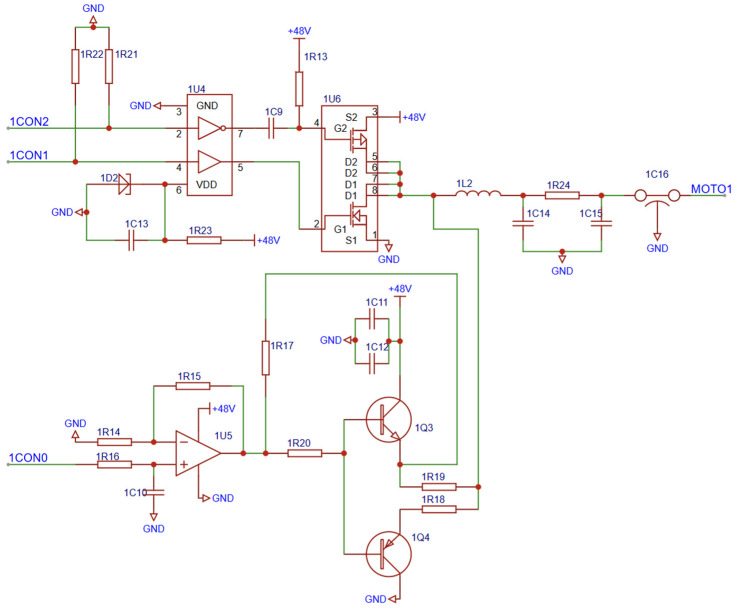
Schematic diagram of the motor drive circuit.

The motor drive module employs the TC4428 chip as the core component of the half-bridge driving circuit, enabling control of the motor’s forward and reverse rotation based on the input signal [23]. The frequency PWM signal is first amplified by an operational amplifier and subsequently processed through a push–pull output stage to enhance the current. An RC low-pass filter smooths the signal to output the final driving waveform. The schematic diagram of the motor drive circuit is shown in Figure 4.

The motor drive circuit features an operational amplifier-based power amplification design comprising an input stage, an operational amplifier power amplification stage, and a push–pull output circuit. Since the motor is driven by a sawtooth wave, the PWM signal with a gradually varying duty cycle is rectified into a sawtooth waveform, which serves as the motor’s driving source. The operational amplifier’s power amplification stage boosts this low-power signal to a high-power output suitable for driving the motor. The simplicity and low cost of the operational amplifier make it an ideal choice for driving the low-power ultrasonic motor employed in this study.

To meet the motor’s drive voltage requirement of 48 V, the input signal is amplified to the same level. For this purpose, the MC33171D operational amplifier from STMicroelectronics is used, and an RC low-pass filter is integrated at the input to mitigate the frequency noise during the signal transmission.

A unipolar half-bridge driving circuit is employed to control the motor’s forward and reverse rotations by adjusting the switching timing of the upper and lower bridge arms via the PWM [24]. This circuit comprises two power switching devices (e.g., MOSFETs or IGBTs), a unipolar DC power supply, and the associated control circuitry.

The two switching devices operate alternately to control the voltage applied across the motor terminals. When the upper bridge arm switch is active, a positive voltage is applied to one end of the motor while the other end is grounded. When the lower bridge arm switch is active, both ends of the motor are grounded, resulting in zero voltage. By varying the duty cycle between the upper and lower bridge arms, the motor’s direction of rotation can be controlled.

The unipolar power supply design offers simplicity and stability, eliminating the need for a negative power source. The PWM control scheme enables the straightforward implementation of the forward and reverse motor motions while maintaining a relatively simple circuit structure [25]. Additionally, since only positive voltage is applied, motor losses are reduced, leading to improved efficiency. This configuration is particularly suitable for the low-power motor driving requirements of this experiment.

**Figure 5:**
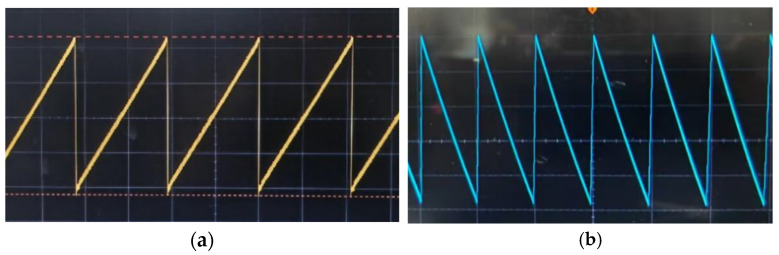
Diagram of the drive circuit output signal. (**a**) shows the sawtooth wave signal during the motor’s forward rotation, with a peak-to-peak value of 45.3 V and a frequency of 4 kHz. (**b**) illustrates the sawtooth wave signal during the motor’s reverse rotation, with a peak-to-peak value of 45 V and a frequency of 2 kHz. The stepping mode and the direction of the motor can be adjusted using a button or via serial communication, with step sizes of 2 kHz for large steps and 4 kHz for small steps.

The output signal detection experiment processes the frequency square wave signal with a variable duty cycle through the hardware circuit, generating the sawtooth waveform shown in Figure 5.

### 2.3. Feedback Design

**Figure 6:**
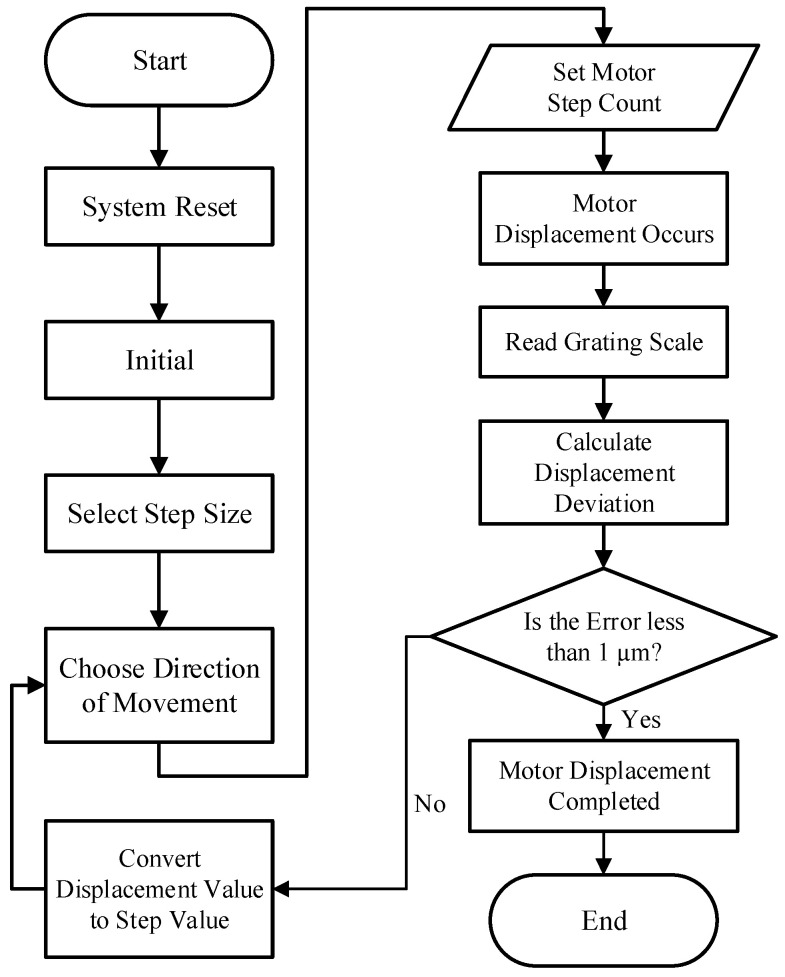
Program control flowchart.

In precision control applications involving ultrasonic motors, a grating scale is employed as a high-precision displacement measurement device, providing real-time feedback on the motor’s position [26]. The program control flowchart of the system is shown in Figure 6.

**Figure 7:**
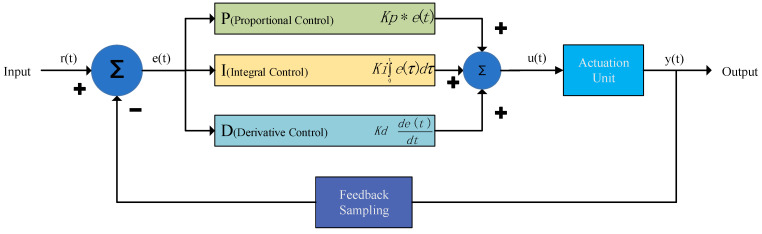
Block diagram of the PID controller system.

This study utilizes a PID algorithm to implement a closed-loop feedback system integrating the ultrasonic motor and the grating scale sensor. This setup ensures that the ultrasonic motor can rapidly and accurately move to the specified position. The system collects real-time displacement data from the grating scale, which is then transmitted to the microcontroller (MCU) for processing. The MCU is preconfigured with three key PID parameters—proportional, integral, and derivative—which are used to calculate the motor’s drive signal. Upon receiving the position information from the grating scale, the MCU compares the target position with the actual position to compute the position error [27]. Using the PID algorithm, the MCU generates a control signal that adjusts the motor’s drive voltage to achieve precise control of its position [28] (Figure 7).

In the PID control scheme, the proportional part (*P*) responds to position errors, providing a quick corrective action. The integral term (*I*) addresses accumulated steady-state errors, while the derivative term (*D*) predicts the system dynamics, contributing to enhanced stability. This combination not only ensures that the grating scale monitors the motor’s position in real time but also enables the MCU to implement closed-loop control, allowing the ultrasonic motor to achieve accurate positioning under various load conditions.

$$u(t)=Kp[e(t)+\frac{1}{Ti}\int_{0}^{t}e(t)dt+Td\frac{de(t)}{dt}]$$

As a traditional PID algorithm is based on continuous-time functions, its direct implementation in digital systems is impractical. Therefore, the algorithm is discretized to enable its application within the MCU. By converting the PID algorithm into a discrete form, the proportional, integral, and derivative components are calculated independently during each sampling period. Starting at *t* = 0, data sampling occurs at regular intervals of t, producing a sequence of discrete time points:

$$\left(e_{0},e_{1},e_{2},e_{3}\ldots e_{k}\right)\;\left(u_{0},u_{1},u_{2},u_{3}\ldots u_{k}\right)$$

The discrete form of the PID algorithm is derived as follows:

$$u(k)=Kp*e(k)+\frac{Kp*T}{Ti}*\sum_{j=1}^{k}e(j)+\frac{Kp*Td}{T}*[e(k)-e(k-1)]$$

where *Kp* is the proportional gain coefficient, *Ti* is the integral time constant, *Td* is the derivative time constant, *T* is the sampling interval, *u*(*t*) is the PID controller output, and *e*(*t*) is the difference between the setpoint *r*(*t*) and the measured value.

By discretizing the PID function, the computation process is simplified and the system’s responsiveness is enhanced. The discretized values make the implementation in the microcontroller unit (MCU) more intuitive and efficient, thereby improving the accuracy and reliability of the ultrasonic motor control process.

## 3. Ground Strain Observation

In the field of terrain deformation observation, the devices used to measure small displacements require laboratory calibration prior to installation. The SS-Y type extensometer employed in this study requires a strain resolution of better than 5 × 10^−10^ strain and a linearity not exceeding 1%. Therefore, during testing, it is essential that the sensor’s linearity and resolution meet these specifications.

**Figure 8:**
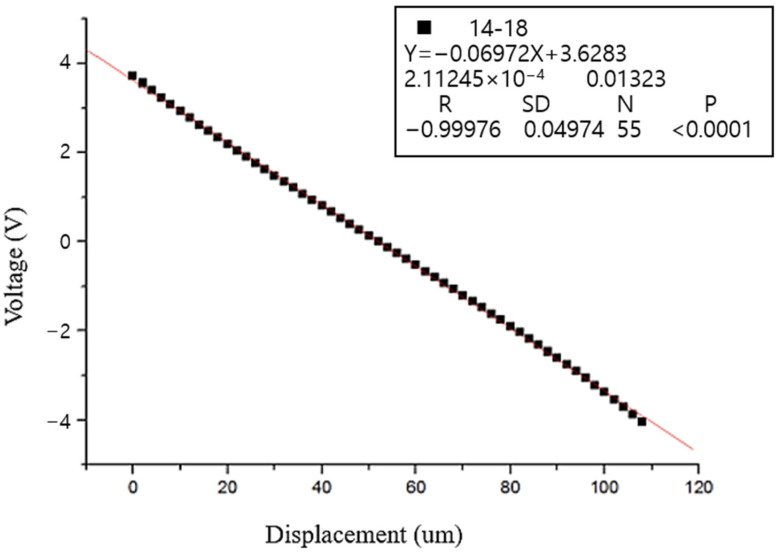
Calibration curve diagram.

In the laboratory, a micro-positioning stage is used to calibrate the capacitive sensor. The micro-positioning stage moves in increments of 10 µm to measure the output voltage of the sensor, enabling the determination of the sensor’s linearity (Table 2 and Figure 8).

Based on the test data, the linearity of the sensor was calculated to be 0.024%, which satisfies the required linearity specification.

A repeatability test was conducted in the laboratory to assess the consistency of the sensor calibration. Using AT commands, calibration commands were sent to the strain gauge. A step size of 30 µm was used for the calibration. Each calibration session involved stabilizing the instrument for 60 s prior to calibration, followed by another 60 s waiting period for data stabilization before the readings were taken. After the motor returned to its initial position, it was allowed to stabilize for an additional 60 s before proceeding to the next calibration. A total of five calibrations were performed, and the repeatability of the calibration was calculated based on the results. These results are presented in Table 3.

From the calculated data, the sensor calibration repeatability was found to be 0.06%, which meets the standards for terrain deformation observation.

**Figure 9:**
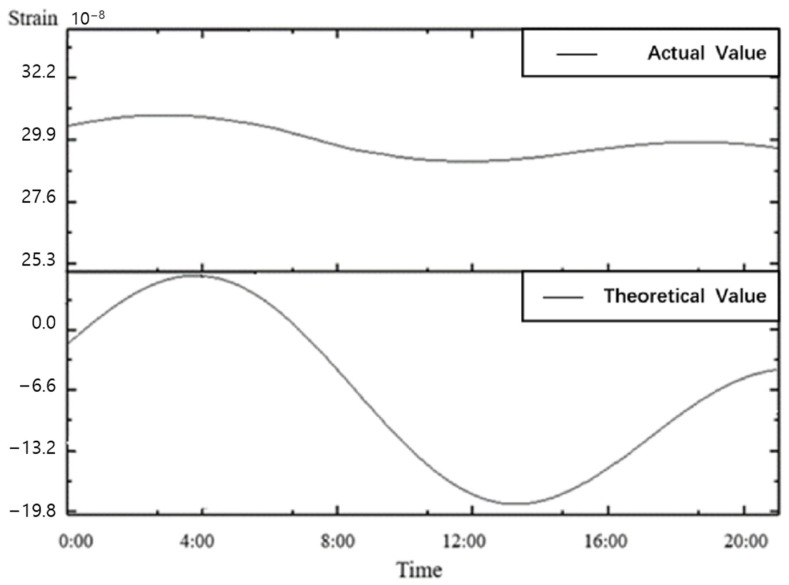
Theoretical values and observed values curve for Chongli Station on 1 February 2024.

Figure 9 shows the theoretical values and observed values for Chongli Station on 1 February 2024. The trough around 13:00 was used for the calculation. According to the seismic observation instrument network technology standard DB/T31.2-2008 [29], the resolution was determined to be 2.0 × 10^−11^ strain, which satisfies the resolution requirements (Table 4).

Field observations were conducted in January 2024 at the seismic station in Chongli County, Zhangjiakou City, Hebei Province using the ultrasonic-motor-based full-range capacitive sensor. During the testing period, the extensometer was installed on two side piers, with the sensor oriented 19° east of north. After the installation, clear solid tidal observations were promptly recorded and the observation curve demonstrated smooth, consistent data.

**Figure 10:**
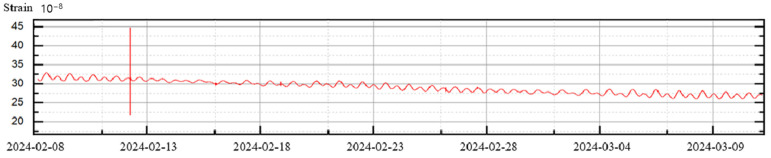
Measured data curve of the extensometer at Chongli Seismic Station.

Figure 10 displays the solid tidal curve recorded by the extensometer, providing continuous observation data from 8 February 2024 to 9 March 2024. A magnitude 6.1 earthquake originating from the Japanese volcanic island region (latitude 22.05° N, longitude 142.85° E) with a focal depth of 250 km was also recorded during this period.

## 4. Discussion

**Figure 11:**
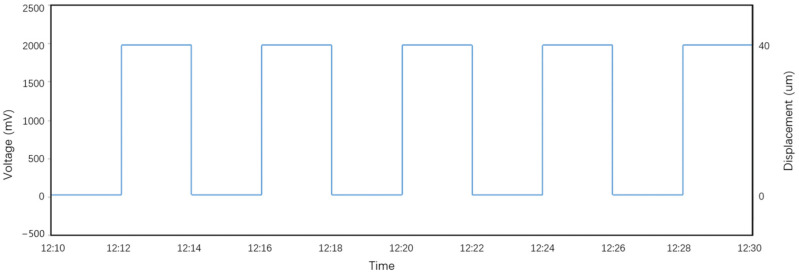
Calibration curve of the SSY extensometer based on the eddy current sensor.

The types of extensometers are quite diverse. Currently, the SSY extensometer, which uses an eddy current sensor, is widely used in geophysical networks. The SSY extensometer employs a micro-displacement platform composed of a variable-speed motor and a worm gear as its zeroing mechanism, allowing for zeroing operations over a larger range and extending its measurement capacity. However, due to the lower positioning accuracy of the zeroing mechanism, a separate calibration mechanism has been established. This calibration mechanism consists of a precision micro-displacement calibration platform based on the principle of inclined wedge block displacement transfer. During calibration, the device is calibrated with a step size of 40 µm, and the output voltage change of the extensometer during each calibration is approximately 2000 mV (output voltage ±2000 mV), using single-step repeated calibration. Figure 11 shows the calibration data and calibration curve for the SSY extensometer using the eddy current sensor.

**Figure 12:**
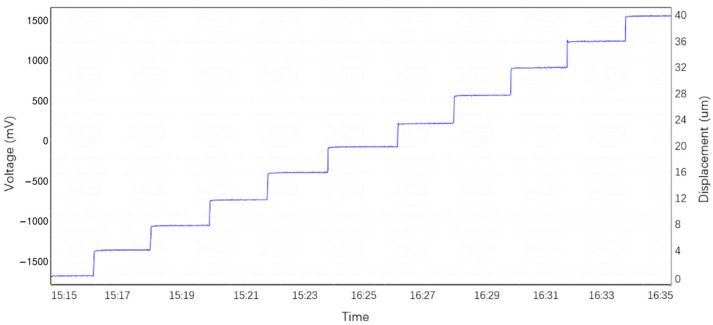
Linear calibration curve of the full-range capacitive sensor extensometer based on the ultrasonic motor.

Capacitive sensors have a higher sensitivity compared to eddy current sensors, allowing for strain measurement accuracy of up to 10^−10^ over shorter measurement bases. The measurement baseline of the SSY extensometer is generally between 10 and 30 m, while extensometers using capacitive sensors can achieve a minimum measurement baseline of 0.1 m. Additionally, the extensometer designed in this study uses an ultrasonic motor for zeroing and range extension, achieving a positioning accuracy of 0.1 µm, making it suitable for direct calibration. During calibration, it typically moves continuously in one direction with a step size of 4 µm, allowing for full-range linear calibration. Figure 12 shows the linear calibration curve of the full-range capacitive sensor extensometer based on the ultrasonic motor.

This study compares the measured data from the SSY extensometer based on an eddy current sensor at Hohlashan Station in Korla, Xinjiang, with the measured data from the designed extensometer at Chongli Station in Hebei.

**Figure 13:**
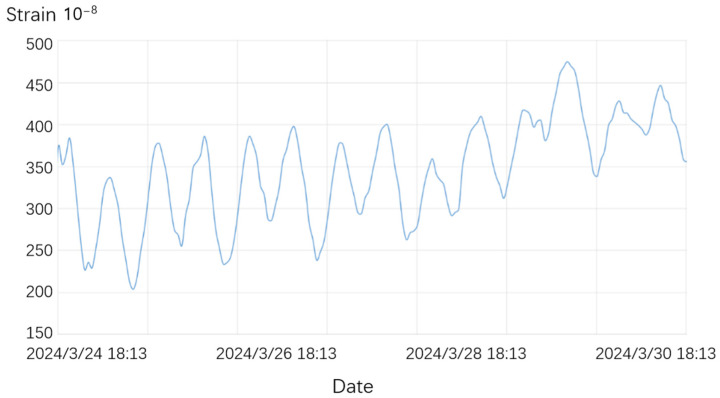
Measured data curve of the SSY extensometer at Hohlashan Station, Korla, Xinjiang.

**Figure 14:**
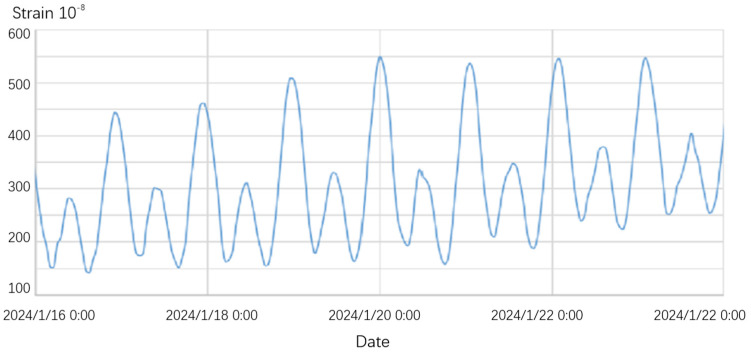
Measured data curve of the full-range capacitive sensor extensometer based on the ultrasonic motor at Chongli Station, Hebei.

The data from Hohlashan Station in Xinjiang cover the east–west direction from 24 March to 30 March 2024, while the data from Chongli Station in Hebei cover the north–south direction from 16 January to 24 January 2024, as shown in Figure 13 and Figure 14. Both datasets recorded clear solid tidal signals, with the observation curves reflecting smooth and distinct solid tides.

This study introduces a novel full-range capacitive sensor extensometer based on high-precision ultrasonic motors. The extensometer uses a capacitive sensor as the measuring device, significantly enhancing the measurement accuracy. By precisely controlling the displacement of the sensor plates with the ultrasonic motor, the capacitive sensor remains within its linear region, thereby extending the measurement range. This approach ensures that the extensometer can provide high-precision measurements without exceeding its operational limits, thereby prolonging the service life of the measurement structure and enhancing the overall performance of the instrument.

The field tests conducted at the Chongli Seismic Station demonstrated that the device achieved a resolution better than 5 × 10^−10^ strain and linearity better than 1%. These results facilitated clear observations of the solid tidal and coseismic response variations. Additionally, the harmonic analysis of the solid tidal data met the stringent requirements for geophysical field observations, confirming the reliability and adaptability of the device in complex geological environments. Throughout the testing process, the device exhibited high reliability, strong adaptability, and ease of operation, enabling rapid deployment without the need for specialized technical personnel. This feature highlights the significant potential of the extensometer for geophysical terrain deformation monitoring, providing reliable data to support related research and advancing the development and application of geological monitoring technologies.

## 5. Conclusions

1. This paper provides a detailed description of the hardware circuit design based on high-precision ultrasonic motors, offering a solution for the effective integration of the full-range capacitive sensor. This design allows for more precise control of the displacement of the sensor plates, thereby enhancing the measurement sensitivity and accuracy. In the system design, a grating scale is used as a closed-loop feedback mechanism, supplemented by a PID algorithm, which improves the stability and response speed of the control system, ensuring high precision and reliability throughout the measurement process.
2. The telescopic instrument based on the full-range capacitive sensor and ultrasonic motor exhibits significant advantages, including high measurement accuracy and full-range measurement capabilities. This instrument maintains high linearity across a wide dynamic measurement range. Moreover, the rapid response characteristics of the ultrasonic motor enable the device to promptly capture instantaneous large-scale geological changes (such as coseismic deformations caused by earthquakes), thereby enhancing the real-time monitoring capability and ensuring high reliability.
3. The telescopic instrument has been field-tested at the Chongli Seismological Station, with results showing a resolution better than 5 × 10^−10^ strain and linearity better than 1%. The device can clearly observe changes in solid tides and coseismic responses. These performance metrics indicate that the telescopic instrument has significant application potential in geophysical deformation monitoring, providing reliable data support for related research and advancing the development and application of seismic monitoring technology.

## Figures and Tables

**Table 1:** Performance comparison of different types of micro-displacement sensors.

Sensor Type	Capacitive Micro-Displacement Sensor	Eddy Current Displacement Sensor	Differential Transformer Displacement Sensor
Measurement principle	Based on capacitance changes as electrode displacement varies	Based on eddy current effects; proximity of metal changes inductance	Electromagnetic induction; measured object affects secondary coil EMF
Measurement range	Micrometers to several millimeters; sub-micrometer accuracy for smaller ranges	Micrometers to tens of millimeters	Several millimeters to hundreds of millimeters
Accuracy	Sub-micrometer level	Micrometer level	Micrometer level
Sensitivity	High; measures displacement changes at sub-micrometer level	Relatively high	Relatively high
Advantages	High accuracy, robust against electromagnetic interference, fast response	Larger range, low environmental sensitivity	Wide range, strong interference resistance

**Table 2:** Relationship between displacement values and sensor output voltage.

Microstage displacement (μm)	0	10	20	30	40	50
Sensor output (V)	3.725	2.903	2.135	1.403	0.698	0.001
Microstage displacement (μm)	60	70	80	90	100	110
Sensor output (V)	−0.69	−1.399	−2.128	−2.894	−3.715	4.536

**Table 3:** Calibration repeatability test data.

Start voltage (mV)	837.47	835.12	835.08	830.6	824.25
Post-calibration voltage (mV)	−1308.3	−1311.38	−1312.91	−1317.6	−1321.08
Calibrated value (mV)	2145.77	2146.5	2147.99	2148.2	2145.33

**Table 4:** Resolution calculation table for the solid tidal strain gauge.

N	Time	Theory Value	Time Interval	Obs Value	Norm Value	Fit Value	Difference
		d_i_	T_i_	d_i_′	d_i_″	$\bar{{d_{i}}^{\prime \prime }}$	Δd_i_″
		10^−10^	min	10^−10^	10^−10^	10^−10^	10^−10^
−7	13:23	3299.65	20	1,938,576.40	20,050.78	20,031.15	19.6266
−6	13:22	3301.36	19	1,938,254.40	20,047.45	20,033.90	13.5514
−5	13:21	3303.00	18	1,938,328.60	20,047.45	20,036.64	10.8064
−4	13:19	3306.00	16	1,937,961.40	20,044.42	20,039.39	5.0308
−3	13:17	3308.65	14	1,937,579.10	20,040.47	20,042.13	1.6685
−2	13:14	3311.98	11	1,937,272.30	20,040.47	20,044.88	4.4135
−1	13:11	3314.53	8	1,936,928.50	20,033.74	20,047.62	13.8878
0	13:03	3317.50	0	1,936,697.90	20,031.35	20,050.37	19.0175
1	12:55	3314.89	8	1,936,437.90	20,031.35	20,047.62	16.2725
2	12:51	3311.49	12	1,936,103.60	20,025.20	20,044.88	19.6750
3	12:48	3308.03	15	1,935,925.30	20,023.36	20,042.13	18.7733
4	12:46	3305.29	17	1,935,894.70	20,023.36	20,039.39	16.0283
5	12:44	3302.20	19	1,937,020.60	20,034.69	20,036.64	1.9548
6	12:43	3300.53	20	1,937,464.00	20,039.28	20,033.90	5.3766
7	12:41	3296.92	22	1,937,699.90	20,039.28	20,031.15	8.1216
k	0.010343044				Δd″max × k	0.2035	

## Data Availability

All data are contained within the article.
